# High-Content Genome-Wide RNAi Screen Reveals *CCR3* as a Key Mediator of Neuronal Cell Death

**DOI:** 10.1523/ENEURO.0185-16.2016

**Published:** 2016-10-24

**Authors:** Jianmin Zhang, Huaishan Wang, Omar Sherbini, Emily Ling-lin Pai, Sung-Ung Kang, Ji-Sun Kwon, Jia Yang, Wei He, Hong Wang, Stephen M. Eacker, Zhikai Chi, Xiaobo Mao, Jinchong Xu, Haisong Jiang, Shaida A. Andrabi, Ted M. Dawson, Valina L. Dawson

**Affiliations:** 1 Neuroregeneration Program, Institute for Cell Engineering, Johns Hopkins University School of Medicine, Baltimore, MD 21205; 2 Department of Neurology, Johns Hopkins University School of Medicine, Baltimore, MD 21205; 3 Department of Immunology, Neuroscience Center, Institute of Basic Medical Sciences, Chinese Academy of Medical Sciences and School of Basic Medicine, Peking Union Medical College, State Key Laboratory of Medical Molecular Biology, Beijing, 100005, China; 4 Solomon H. Snyder Department of Neuroscience, Johns Hopkins University School of Medicine, Baltimore, MD 21205; 5 Department of Physiology, Johns Hopkins University School of Medicine, Baltimore, MD 21205; 6 Department of Pharmacology and Molecular Sciences, Johns Hopkins University School of Medicine, Baltimore, MD 21205

**Keywords:** excitotoxicity, ischemia, library screen, stroke

## Abstract

Neuronal loss caused by ischemic injury, trauma, or disease can lead to devastating consequences for the individual. With the goal of limiting neuronal loss, a number of cell death pathways have been studied, but there may be additional contributors to neuronal death that are yet unknown. To identify previously unknown cell death mediators, we performed a high-content genome-wide screening of short, interfering RNA (siRNA) with an siRNA library in murine neural stem cells after exposure to *N*-methyl-*N*-nitroso-*N*′-nitroguanidine (MNNG), which leads to DNA damage and cell death. Eighty genes were identified as key mediators for cell death. Among them, 14 are known cell death mediators and 66 have not previously been linked to cell death pathways. Using an integrated approach with functional and bioinformatics analysis, we provide possible molecular networks, interconnected pathways, and/or protein complexes that may participate in cell death. Of the 66 genes, we selected *CCR3* for further evaluation and found that CCR3 is a mediator of neuronal injury. CCR3 inhibition or deletion protects murine cortical cultures from oxygen-glucose deprivation–induced cell death, and *CCR3* deletion in mice provides protection from ischemia in vivo. Taken together, our findings suggest that CCR3 is a previously unknown mediator of cell death. Future identification of the neural cell death network in which CCR3 participates will enhance our understanding of the molecular mechanisms of neural cell death.

## Visual Abstract

**Figure:**
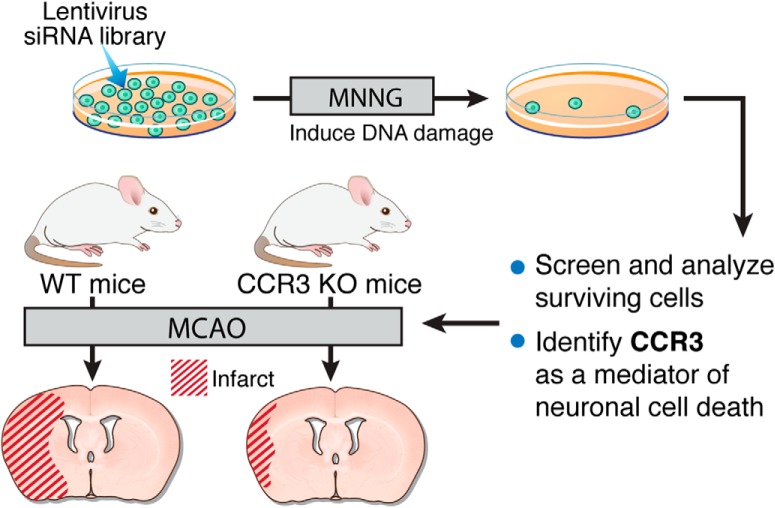

## Significance Statement

A high-content siRNA genome-wide screen identified 80 mediators of cell death in neural stem cells in response to DNA damage injury. These cell death mediators possibly form a molecular network containing several partially overlapping and interconnected pathways or protein complexes. Through *in vitro* and *in vivo* experiments, we confirmed that one of the identified molecules, CCR3, is a mediator of neuronal cell death. Further investigation of the mechanisms of CCR3 action in neuronal injury is warranted. Additionally, the other 65 genes may provide new opportunities to understand and prevent neuronal cell death.

## Introduction

Neuronal cell death plays an important role in the proper development of the immature nervous system, but in the adult, neuronal cell death underpins neuronal dysfunction caused by disease, trauma, or ischemic injury. Although neuronal cell death in the context of nervous system development is prominently triggered by the absence of cell survival factors and induction of apoptosis, death of mature neurons in the context of neurological disease is induced through the activation of a variety of cell death mediators and their signaling networks (Becker and Bonni, 2004). Although many genetic screens are conducted in smaller organisms such as *Drosophila* or *Caenorhabditis elegans*, recently RNA-mediated interference (RNAi) loss-of-function experiments have been applied to genome-wide screens in mammalian cells to identify genes that are required for a wide variety of specific phenotypes (Kittler et al., 2004; Paddison et al., 2004; Moffat et al., 2006; Hitomi et al., 2008). Because DNA damage is a contributing factor in neuronal cell death after stroke or in neurodegenerative diseases, to identify currently unknown mediators of cell death, we performed a genome-wide RNAi screen in neural stem cells after exposure to the DNA alkylating agent, *N*-methyl-*N*-nitroso-*N*′-nitroguanidine (MNNG). MNNG promotes nicks and breaks and is known to activate the enzymatic activity of poly(ADP-ribose) polymerase, resulting in a more necrotic-like rather than apoptotic cell death which has been designated parthanatos. (D’Amours et al., 1999; Zong et al., 2004).

In this study, we performed a genome-wide screen using the mouse GeneNet Mouse 40K FIV lentiviral small, interfering RNA (siRNA) library. We identified 80 key mediators for DNA damage-induced neural cell death. Among these 80 genes, 66 have not been previously been linked to DNA damage response pathways or cell death cascades. After functional analysis and bioinformatics analysis of the gene set, we selected the C-C chemokine receptor 3 (*CCR3*) for further characterization. CCR3 is a seven-transmembrane G protein–linked receptor that has been extensively studied in the setting of inflammation and allergic reaction in peripheral tissues (Willems and Ijzerman, 2010). In addition to expression in bone marrow–derived cells including basophils, eosinophils, and microglia, CCR3 is expressed in astrocytes and neurons in both the developing and adult brain (van der Meer et al., 2000). Through loss-of-function experiments, we confirm that CCR3 functions in cell death signaling in cortical cultures exposed to oxygen-glucose deprivation (OGD) and contributes to ischemic brain injury *in vivo*. Understanding how CCR3 contributes to neuronal loss and further investigation into the potential neuroprotective actions of CCR3 inhibition may reveal new therapeutic opportunities for treatment of ischemic injury or neurodegenerative diseases.

## Methods

### Cell culture and lentiviral siRNA infection

Mouse neural stem cells were cultured and infected with mouse 40K genome-wide lentiviral siRNA provided by System Biosciences (Palo Alto, CA) according to the manufacturer’s manual. Briefly, mouse neural stem cells (ANSO4E) were plated in twelve 10-cm plates at a density of about 5 × 10^5^ cells per plate 24 h before viral infection. On the second day, 1 × 10^7^ infection units of mouse 40K genome-wide lentivirus siRNA was thawed and diluted in 30 ml complete neural cell culture medium (20 ng/ml basic fibroblast growth factor, 20 ng/ml epidermal growth factor in Dulbecco’s modified Eagle’s medium/F-12 medium) with 5 μg/ml polybrene. Ten plates of cultured mouse neural cells were infected with 3-ml viral dilutions (ratio of virus and cell of about 1:5). For the mock transduction control, 3 ml Dulbecco’s modified Eagle’s medium with polybrene was added. All cells were incubated at 37°C with 5% CO_2_. For each plate, the culture medium was removed and replaced with 10 ml fresh complete medium (without polybrene) 12 h after infection. At d 4, the cells were split 1:3 and returned to culture for another 24 h. On d 5 (72 h after transduction), neural stem cells were treated with 500 μm MNNG for 15 min, washed two times with medium, and incubated at 37°C with 5% CO_2_ for 3 d. The dead cells were removed by changing the medium every 3 d, and the surviving cells were grown and treated with 500 μm MNNG for secondary selection. The surviving cells were harvested after growing to 10 million cells.

### Recovery of siRNA inserts

When integrated into genomic DNA, the pFIV constructs produced an alternative transcript from that of a fusion of the marker genes (copGFP or Puro) with the siRNA sequence. This alternative transcript was used as a template to amplify the siRNA insert. Recovery of siRNA inserts was performed according to the manufacturer’s instructions. Briefly, total RNA of surviving cells or reference control cells was isolated with TRIzol Reagent (Invitrogen, San Diego, CA) and purified further with RNeasy minicolumns (Qiagen, Valencia, CA). For each sample, 10 μg total RNA in 15 μl RNA-free water was used for cDNA synthesis as recommended by the manufacturer. The primer sequence for reverse transcription was TGCATGTCGCTATGTGTTCTGGGA. Amplification of the inserts from synthesized cDNA required two rounds of PCR. The primers for the first round of PCR were forward, 5′-AATFTCTTTGGATTTGGGAATCTTA-3′, and reverse, 5′-AAAAGGGTGGACTGGGATGAGT-3′. During the second round of PCR with two nested primers (nested forward primer 5′-Bio-ATCGTCAATCACCTTCCTGTCAGA-3′ and nested reverse primer 5′-AATAGAAAGAATGCTTATGGACGCTA-3′), the amplified siRNA targets were labeled with biotin. Biotinylated siRNA targets (15 μg) were hybridized with Affymetrix GeneChip Mouse Genome 430 2.0 Array (#900495; Affymetrix, Santa Clara, CA) to identify surviving cell-associated siRNAs using the manufacturer’s protocols and recommended reagents.

### Individual siRNA knockdown and OGD

Lentiviral siRNA vectors for 17 genes were obtained from Invitrogen, and lentiviruses were individually prepared as described previously (Wang et al., 2011). Primary cultured mouse cortical neurons were cultured and infected with lentiviral siRNAs at 7 days *in vitro*. Lentiviral siRNAs for DsRed were used as control. Seven days after infection, neurons were treated with lethal OGD (90 min) to induce neural cell death. Twenty-four hours after OGD treatment, cell viability was determined by staining with Hoechst 33342 and propidium iodide. Images were captured by charge-coupled device camera under Axiovert 200M fluorescence microscope, and dead and viable cells were counted by a blinded observer using unbiased computer-assisted cell counting software (Axiovision 4.3; Zeiss, Oberkochen, Germany). Cell death rate was determined as the ratio of dead cells to total cells. Three separate individual experiments were performed using three separate wells.

### Middle cerebral artery occlusion

CCR3^–/+^ mice were crossed to generate CCR3^–/–^ mice and wild-type (WT; CCR3^+/+^) mice in the same litters to ensure the same genetic background. We used only the males for middle cerebral artery occlusion (MCAO) to avoid the possibility of sex-specific sensitivity to cell death. Thirteen male CCR3 KO mice and nine WT littermates were subjected to MCAO as described previously (Andrabi et al., 2011). In brief, mice were anesthetized with 1.5–2% isoflurane and maintained at normothermic temperature. Occlusion was performed by placing a monofilament to the base of the middle cerebral artery. The whole MCAO procedure was monitored by laser-Doppler flowmetry with a probe placed on thinned skull over the lateral parietal cortex. After 60 min of occlusion, the filament was removed and the reperfusion was verified. Twenty-four hours later, animal brains were coronally sectioned into five 2-mm-thick sections in a mouse brain matrix and stained in 2% 2,3,5-triphenyltetrazolium chloride solution before fixation in 10% formalin overnight. The infarction area was imaged with a digital camera and analyzed using ImageJ (National Institutes of Health, Bethesda, MD) by an operator blinded to genotype. The infarction volume was calculated by summing the infarct volumes of sections. Infarct size was calculated and expressed as a percentage by using the following formula: (contralateral volume – ipsilateral undamaged volume) × 100/contralateral volume, to eliminate effects of edema as described previously (Kishimoto et al., 2010). The investigator performing the surgery and analyzing infarct size was unaware of the genotype of the mouse.

### Assessment of neurobehavioral activity

Spontaneous motor activities of mice were recorded for 5 min in an animal cage at 24 h, 3 d, and 7 d after MCAO as described previously (Andrabi et al., 2011). Briefly, neurological deficits were assessed on a scale of 0–4 (0, no neurological deficit; 4, severe neurological deficit) according to the criteria: 0 = normal, the mice explored the cage environment and moved around in the cage freely; 1 = the mice could hesitantly move in the cage but did not approach all sides of the cage; 2 = the mice showed postural and movement abnormalities and had difficulty approaching all walls of the cage; 3 = the mice with postural abnormalities tried to move in the cage but did not approach one wall of the cage, and 4 = mice were unable to move in the cage and stayed at the center. The observer was blinded to the genotypes of the animals.

### Data analysis

The software provided with the library on the Data Analysis Program and Gene List was used to analyze the hybridization data and create a report file in a format compatible with common spreadsheets (Excel and .txt file), and further analysis was performed in R. The file lists the intensities of signal, which correspond to the abundance level for each of the specific siRNA species in the library. The data for basic bioinformatics was analyzed with Gene Set Enrichment Analysis (GSEA) and the Eukaryotic Subcellular Localization Database coupled with R. Analysis for gene networks and pathways was performed with Cytoscape v3.3.0 software with GeneMANIA (http://www.genemania.org) plugin according to the instruction manual.

### Statistical analysis

All experiments were repeated at least three times, and quantitative data are presented as the mean ± SEM as calculated by GraphPad Prism6 software (Instat; GraphPad, San Diego, CA). Statistical significance was assessed by one-way ANOVA. Significant differences were identified by post hoc analysis using the Tukey–Kramer post hoc method for multiple comparisons. Assessments were considered significant with *p* < 0.05 and nonsignificant with *p* > 0.05.

## Results

### Genome-wide screen of mediators for DNA damage–induced neural cell death

**Figure 1.**
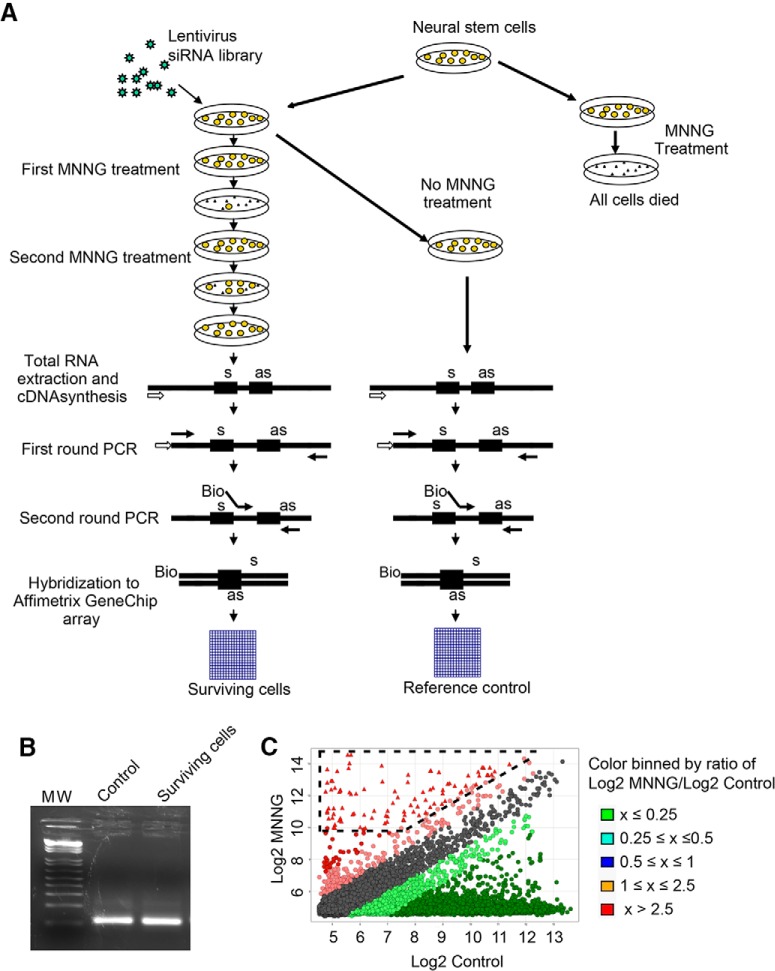
siRNA screening for genes required for DNA damage–induced neural cell death. ***A***, Schematic diagram of lentiviral siRNA library screening. ***B***, Fragments of siRNA inserts were amplified from lentivirus-infected, MNNG-treated surviving neural stem cells and reference control cells (lentivirus infection but no MNNG treatment) by two rounds PCR. ***C***, Scatterplot analysis of siRNA inserts enriched in neural stem cells treated by MNNG.

Mouse adult neural stem cells were transduced with the mouse 40K genome-wide FIV lentiviral siRNA library from System Biosciences (Fig. 1*A*). This lentiviral siRNA library contains 150,000 siRNA sequences that target 39,000 mouse mRNA transcripts with at least four different siRNA sequences for each target mRNA. We used a multiplicity of infection of ∼0.2 to ensure that each cell received a single lentiviral-encoded siRNA. The cell cultures were exposed to 500 μm MNNG treatment for 15 min, which in nontransduced cultures results in 100% lethality 24 h after exposure (data not shown). In FIV lentiviral siRNA–infected cultures, surviving neural stem cells were grown and expanded and subjected to a second treatment with MNNG (Fig. 1*A*). Surviving neural stem cells were grown and expanded, followed by extraction of mRNA. The siRNA templates were recovered by PCR using primers that recognize common sequences within the siRNA templates. A second PCR using nested primers with a biotin residue at the 5′ end and another containing a 5′-phosphate group were used to identify the siRNA inserts by hybridizing to the GeneChip Mouse Genome 430 2.0 Array (Fig. 1*B*,*C*; Huaux et al., 2005). After hybridization, the image of the processed chip was scanned, and data were subjected to quantile normalization and ANOVA. We selected 80 genes with signal intensities of more than 1000 and log2 ratios between surviving cells and reference controls (neural stem cells transduced with the library but not treated with MNNG) of more than 2.5 (**Table 1**). Of these 80 genes, 14 (17.5%) are known mediators of cell death, whereas the others have not been previously linked to DNA damage–induced cell death or other cell death pathways.

**Table 1.** Identified genes required for DNA damage–induced neural cell death

Category and gene symbol	Full gene name	GenBank accession no.	Control	MNNG	Log2 ratio
Cell death					
*Dedd*	Death effector domain-containing	AK006814	937	9842	3.39
*Eif2ak2*	Eukaryotic translation initiation factor 2 alpha kinase 2	BE911144	3	7879	11.35
*Fasl*	Fas ligand (TNF superfamily, member 6)	NM_010177	304	3395	3.48
*Trim39*	Tripartite motif-containing 39	NM_024468	480	4511	3.23
*CCR3*	Chemokine (C-C motif) receptor 3	NM_009914	770	4474	2.54
*NME1*	Non-metastatic cells 1, protein (NM23A) expressed in	AV156640	94	1710	4.19
*GAS6*	Growth arrest-specific 6	NM_019521	4	7793	10.93
*VAMP3*	Vesicle-associated membrane protein 3	BE994144	578	7037	3.61
*NCOA1*	Nuclear receptor coactivator 1	BE996469	1242	9787	2.98
*IFI202B*	Interferon activated gene 202B	AV229143	343	12,047	5.13
*STAT3*	Signal transducer and activator of transcription 3	AK004083	907	5156	2.51
*ABCE1*	ATP-binding cassette, sub-family E, member 1	BG063303	344	2480	2.85
*CAPN2*	Calpain 2, (m/II) large subunit	NM_009794	1625	13,981	3.10
*Map3k12*	Mitogen activated protein kinase kinase kinase 12	BB370469	1	1069	9.92
Signal transduction					
*Sgsm1*	Small G protein signaling modulator 1	AK010756	11	5037	8.84
*Grm7*	Glutamate receptor, metabotropic 7	BB539404	21	4723	7.81
*V1ra5*	Vomeronasal 1 receptor, A5	NM_053220	225	3542	3.98
*Tbc1d8b*	TBC1 domain family, member 8B	AK014817	1841	11,784	2.68
*Fgf23*	Fibroblast growth factor 23	AF263536	1344	14,077	3.39
Regulation of transcription					
*Pcgf6*	Polycomb group ring finger 6	BC016195	100	1650	4.04
*Bnc1*	Basonuclin 1	U88064	200	2708	3.76
*Zbtb46*	Zinc finger and BTB domain containing 46	AK016700	91	1220	3.74
*Med30*	Mediator complex subunit 30	NM_027212	312	1818	2.54
*E2f5*	E2F transcription factor 5	BC003220	243	2547	3.39
*Myt1*	Myelin transcription factor 1	NM_008665	661	6287	3.25
1300003B13Rik	RIKEN cDNA 1300003B13 gene	AK004870	161	1427	3.15
*Snap91*	Synaptosomal-associated protein 91	BG068132	1	2092	11.3
*Ccnl1*	Cyclin L1	BB543556	1	2347	11.2
Protein kinase activity					
*Stk25*	Serine/threonine kinase 25 (yeast; Stk25)	BG068951	1	1499	10.55
*Gucy2e*	Guanylate cyclase 2e	NM_008192	1211	11,563	3.26
*Tcp10c*	T-complex protein-10 complete	AV257292	784	4904	2.65
*Stk22a*	Serine/threonine kinase 22A	NM_009435	1	1966	10.94
Oxidoreductase activity					
*Mosc2*	MOCO sulfurase C-terminal domain containing 2	NM_133684	11	5283	8.91
*Cyp2c65*	Cytochrome P450, family 2, subfamily c, polypeptide 65	AK008688	283	4801	4.08
Hydrolase activity					
*Fbp2*	Fructose bisphosphatase 2	NM_007994	3	5351	10.80
*Pgam5*	Phosphoglycerate mutase family member 5	BC021317	4	1533	8.58
*Orc4l*	Origin recognition complex, subunit 4-like	BB620704	27	1877	6.12
*Mtm1*	X-linked myotubular myopathy gene 1	NM_019926	1395	13,912	3.32
Ubiquitin-protein ligase activity					
*March1*	Membrane-associated ring finger (C3HC4) 1	AK013582	1	4915	12.26
Transporter					
*Ubl7*	Ubiquitin-like 7 (bone marrow stromal cell-derived)	BC016456	11	1513	7.1
*Sec15l1*	SEC15-like 1 (S. cerevisiae)	BC026859	471	2855	2.6
*Clic5*	Chloride intracellular channel 5	BB028501	25	1805	6.17
*Slc8a1*	Solute carrier family 8, member 1	BM123508	548	6850	3.64
*Clcc1*	Chloride channel CLIC-like 1	BC003247	164	1391	3.08
Protein complex					
*Mrpl17*	Mitochondrial ribosomal protein L17	BB343967	1	12,983	13.66
*Sfrs8*	Splicing factor, arginine/serine-rich 8	BE688816	13	2396	7.53
*Clptm1*	Cleft lip and palate associated transmembrane protein 1	NM_019649	137	3821	4.8
Metabolism					
*Tssk1*	Testis-specific serine kinase 1	NM_009435	1	1966	10.94
*Ndufb2*	NADH dehydrogenase (ubiquinone) 1 beta subcomplex, 2	NM_026612	19	24,343	10.32
*Spopl*	Speckle-type POZ protein-like	BM116703	436	11,419	4.71
*Mat2a*	Methionine adenosyltransferase II, alpha	BB488978	1521	9954	2.71
Unknown					
*Tollip*	Toll interacting protein	BB400304	10	1376	7.10
*Zmynd19*	Zinc finger, MYND domain containing 19	NM_026021	217	2020	3.22
*ZNF706*	Zinc finger protein 706	AA165749	184	7385	5.33
*Clec14a*	C-type lectin domain family 14, member a	NM_025809	98	1301	3.73
*Dexi*	Dexamethasone-induced transcript	NM_021428	6	5740	9.9
*Rg9mtd2*	RNA (guanine-9-) methyltransferase domain containing 2	BG063557	30	4464	7.22
*Rutbc2*	RUN and TBC1 domain containing 2	AK010756	11	5037	8.84
4833422M21RIK	RIKEN cDNA 4833422M21 gene	AK014752	36	1017	4.82
4930455J16RIk	RIKEN cDNA 4930455J16 gene	AK015470	43	5455	6.99
A630057N01RIK	RIKEN cDNA A630057N01 gene	BB224397	881	6339	2.85
*AFTPH*	Aftiphilin	BG071681	12	1323	6.78
*ANKRD13C*	Ankyrin repeat domain 13C	AV104707	43	1760	5.36
*AU021889*	Expressed sequence AU021889	BG068079	5	1846	8.53
*C2ORF77*	Chromosome 2 open reading frame 77	BE282424	21	16,878	9.65
*D14Abb1e*	DNA segment, chromosome 14, Abbott 1 expressed	BM209908	58	1537	4.73
*COMMD8*	COMM domain containing 8	AV365904	2687	16,102	2.58
*DLX1AS*	Distal-less homeobox 1, antisense	AI325350	168	1755	3.38
*FAM19A1*	Family with sequence similarity 19, member A1	AW121549	84	7580	6.50
*Thrap6*	Thyroid hormone receptor associated protein 6	NM_027212	312	1818	2.54
*MSL1*	Male-specific lethal 1 homolog	AW495537	8	11,950	10.54
*MTG1*	Mitochondrial GTPase 1 homolog (S. cerevisiae)	BC027306	23	4386	7.58
*Nalp9b*	NACHT, LRR and PYD containing protein 9b	BG068754	6	1147	7.58
*Surf6*	Surfeit gene 6	NM_009298	303	1964	2.70
*TMEM203*	Transmembrane protein 203	BC022606	10	4837	8.92
*TSPYL3*	TSPY-like 3 (pseudogene)	BB308532	384	2908	2.92
*Btbd4*	BTB (POZ) domain containing 4	AK016700	91	1220	3.74
*Tssc1*	Tumor suppressing subtransferable candidate 1	BB082634	165	2032	3.62
*Pax6os1*	Pax6 opposite strand transcript 1	BB255007	5	1008	7.66
*Ttc21b*	Tetratricopeptide repeat domain 21B	AW554401	350	12,119	5.11

### Validation of identified genes as mediators of cell death

**Figure 2.**
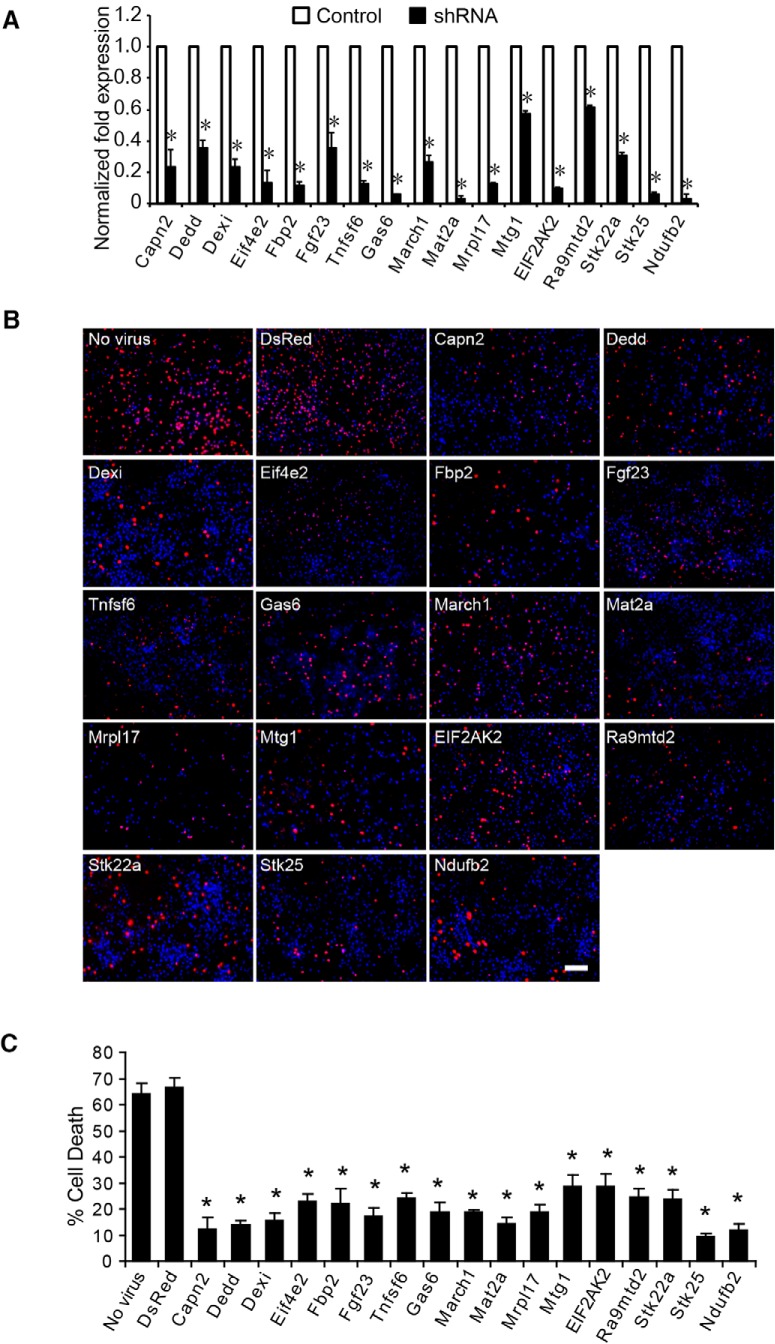
Validation of the siRNA library screening. ***A***, Seventeen genes were randomly selected from the 80 identified in the screen, and expression was knocked down in primary cultured cortical neurons (7 days *in vitro*) by lentiviral siRNAs for the 17 genes. ***B***, Representative images of neurons treated with lethal OGD (90 min) and stained with Hoechst 33342 and propidium iodide (PI). Dead neurons were scored as red cells with condensed or fragmented nuclei. Non-virus-infected neurons were used as viral control, and neurons infected with lentiviral siRNA for DsRed were used as siRNA control. Scale bar =100 µm. ***C***, Quantification of cell death in ***B***. Experiments were performed three times, and data represent the mean ± SEM. Statistical significance from DsRed siRNA control indicated at *p < 0.05; differences between multiple groups were evaluated by one-way ANOVA followed by the Tukey–Kramer post hoc test.

Because the goal of this screen was to identify genes that are unknown cell death effectors that could contribute to neuronal cell death, 17 genes were selected from the 80 identified genes for further analysis in primary mouse cortical neurons exposed to OGD experiments (stroke in a dish). Lentiviral siRNAs for these genes were individually prepared and used to infect primary cultured cortical neurons at 7 days *in vitro*. Four genes (*Capn2*, *Dedd*, *FASLG*, and *EIF2AK2*) that have been previously linked to cell death (Zhang and Ghosh, 2002; Scheuner et al., 2006; Lee et al., 2013; de Oliveira et al., 2014) served as positive controls, and an additional 13 genes that have not been previously linked to cell death were selected at random. Confirmation of gene knockdown was confirmed by reverse-transcription (RT)-PCR (Fig. 2*A*). Nontransduced neurons and neurons transduced with lentiviral siRNA directed against DsRed were used as controls. At day 7 after infection, neurons were subjected to 90 min of OGD, leading to 66.7 ± 3.5% (mean ± SEM) cell death in DsRed siRNA-transduced neurons 24 h after reperfusion with normal media (Fig. 2*B*,*C*). Cell death was significantly and substantially decreased in neurons infected by lentiviral siRNAs for the 17 individual genes (Fig. 2*B*,*C*). These data, taken together, suggest that the genome-wide siRNA library screen was effective in the identification of cell death signaling molecules.

### Biological network analysis of identified genes

**Figure 3.**
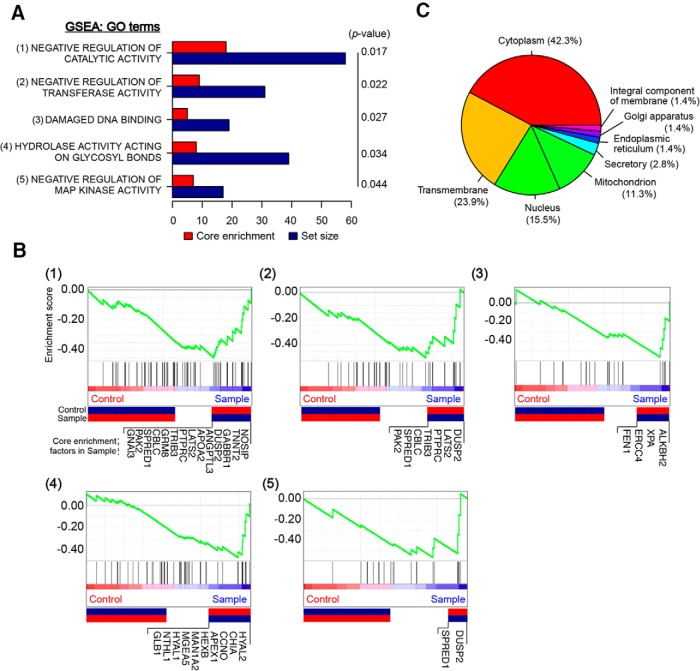
GSEA of identified genes required for neural cell death. The expression data are ranked in the order of differential expression. ***A***, Forty-seven core enrichment factors from the siRNA library screen for DNA damage–induced cell death genes classified by GSEA analysis. ***B***, Five individual plots (*p*-value <0.05) of the running sum for the gene sets in the ranked gene list. ***C***, Subcellular localization was categorized using the top 80 genes from the siRNA library screen. Genes with no annotations were excluded from the analysis.

To ascertain which biological functions maybe associated with the gene set identified in the genome-wide siRNA screen, we used GSEA (Mootha et al., 2003; Subramanian et al., 2005). Five significant gene ontology (GO) terms from microarray were isolated to the DNA damage network (Fig. 3*A*). Of the core enrichment factors in each GO term identified in the siRNA screen, 37 could be classified into GSEA categories as core enrichment factors, and 71 genes from 80 processed signals into subcellular localization categories (Fig. 3*B*,*C*). The primary categories enriched in GSEA analysis mainly include the negative regulation of catalytic activity, negative regulation of transferase activity, damaged DNA binding, hydrolase activity acting on glycosyl bonds, and negative regulation of mitogen-activated protein kinase (MAPK) activity (Fig. 3*A*). We identified 14 core enrichment factors under DNA damage with negative regulation of catalytic activity, seven of which have negative regulation of transferase activity, four with damaged DNA binding, 10 with hydrolase activity acting on glycosyl bonds, and two with negative regulation of MAPK activity (Fig. 3*B*), suggesting that these biological processes are involved in the process of DNA damage–induced neural cell death. Of 71 subcellular localization categories, eight mediators are mitochondrial proteins (Ndufb2, Mrpl17, Mosc2, Snap91, Trim39, Nme1, Abce1, and Mtg1), reinforcing the known role of mitochondria in response to DNA damage injury and cell death (Fig. 3*C*).

### Canonical pathways in DNA damage–induced neural cell death

**Figure 4.**
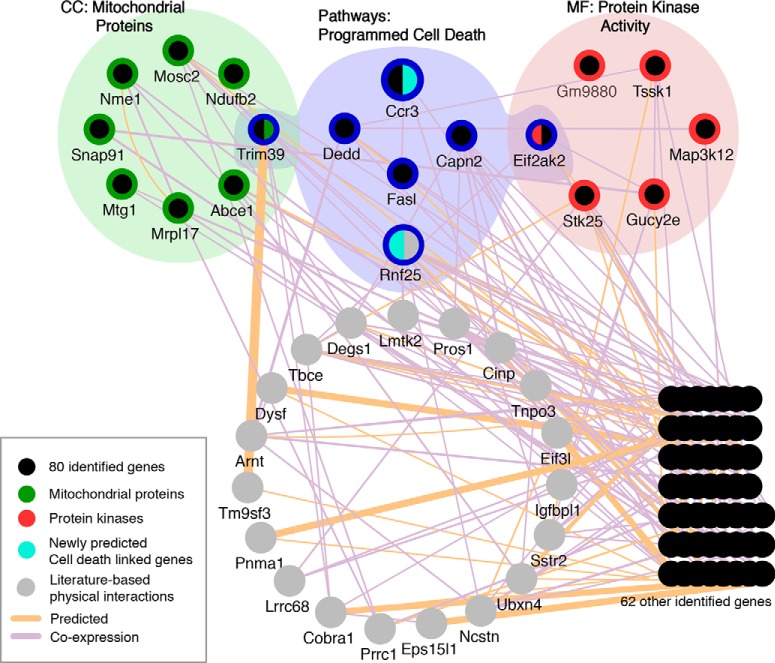
Network analysis of identified genes required for DNA damage–induced neural cell death. Screen results for 80 genes from siRNA library screen and 20 evidence-based predicted genes were mapped onto the network, and highly interconnected clusters containing factors identified from the siRNA library screens were isolated, representing the programmed cell death clusters important for DNA damage–induced neural cell death. A network was initially constructed by Cytoscape v3.3.0 software with GeneMANIA plugin using limited database search for predicted and literature (evidence)-based coexpressed genes. The network was then visually rearranged with combination of GO term results [cellular components (CC) molecular functions (MF), and pathways] from the Database for Annotation, Visualization, and Integrated Discovery and GSEA.

In response to DNA damage, a number of proteins called “DNA damage sensors,” such as protein kinases, are first activated and initiate the DNA damage signal networks, such as the activation of a variety of kinase substrates called “effectors,” leading to programmed cell death (Peng and Chen, 2003; Shiotani and Zou, 2009). To gain insights into the network and pathways of genes identified in this siRNA screen, we used Cytoscape v3.3.0 software with GeneMANIA (http://www.genemania.org) plugin (Shannon et al., 2003; Montojo et al., 2010). A search condition specifies criteria for choosing “predicted” and literature (evidence)-based “coexpressed” genes. Network integrated with GO term results was visually rearranged in Cytoscape (cellular components and molecular functions and pathways from GSEA and the Database for Annotation, Visualization, and Integrated Discovery). Screen results for 80 genes from the genome-wide siRNA library screen and 20 evidence-based predicted genes were mapped onto the network, and highly interconnected clusters containing factors identified from the screens were isolated, thus representing the programmed cell death clusters important for DNA damage–induced neural cell death (**Fig. 4**). From six identified protein kinase activity groups (EIF2AK2, Stk22a, Stk25, Ndufb2, Map3k12, Gucy2e; red colored) and eight mitochondrial proteins (colored green in **Fig. 4**), Eif2ak2 and Trim39 from each group are associated with five other predicted programmed cell death clusters (Ccr3, Dedd, Capn2, FASLG, and Rnf25; colored blue in **Fig. 4**). *RNF25*, a unique gene belonging to the programmed cell death cluster, was generated from the predicted/coexpression network (colored gray; **Fig. 4**). Apart from four validated genes in Figure 2*B*, Ccr3 and Trim39 from screening and RNF25 from prediction are key factors identified for programmed cell death pathways.

### CCR3 is a key mediator of neuronal cell death

**Figure 5.**
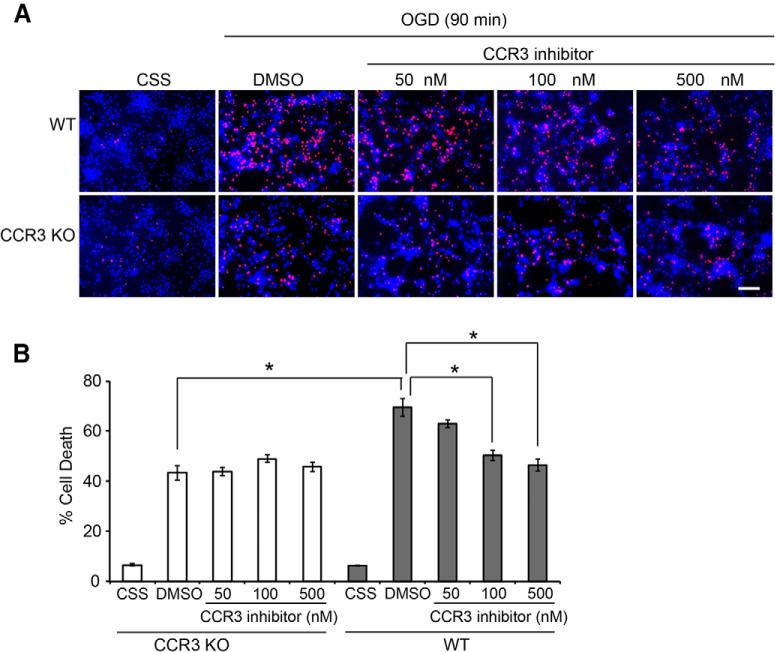
*CCR3* deletion or inhibition protects neurons against OGD-induced excitotoxicity. ***A***, Primary cultured cortical neurons were isolated from CCR3 KO and WT embryos at embryonic day 15. Neurons (14 days *in vitro*) were pretreated with or without CCR3 inhibitor SB328437 at the indicated dose before being subjected to OGD treatment for 90 min. Twenty-four hours after OGD, neurons were stained with Hoechst 33342 and propidium iodide (PI). Dead neurons were scored as red cells with condensed or fragmented nuclei. Scale bar =100 µm. ***B***, Quantification of cell death in different treatments. Experiments were performed at least three times. Data represent the mean ± SEM. * *p*<0.05, one-way ANOVA with Tukey–Kramer test.

Of the many genes identified, *CCR3* is interesting in that it has not been extensively studied in the nervous system. Because of its actions in inflammatory responses and allergic reactions (Willems and Ijzerman, 2010), however, small molecule inhibitors of *CCR3* that are relatively selective and specific have been identified, some of which are currently in clinical trials (Bando et al., 2005; Zhang et al., 2005). To test whether *CCR3* plays a role in neuronal injury, primary cortical cultures were generated from WT and CCR3 knockout (KO) mice. Cultures were exposed to 90 min of OGD in the presence or absence of the CCR3 inhibitor, SB328437 [*N*-(1-naphthalenylcarbonyl)-4-nitro-l-phenylalanine methyl ester; van der Meer et al., 2000], and cell viability was assessed 24 h later. WT cultures exhibited 69.56 ± 3.58% cell death that was markedly reduced by the CCR3 inhibitor at the concentrations of 100 nm (50.41 ± 2.05%) and 500 nm (46.49 ± 2.36%; Fig. 5*A*,*B*). Knockout of *CCR3* also reduced cell death by 37.56%; it was not further reduced by the CCR3 inhibitor, demonstrating the selectivity of the inhibitor (Fig. 5*A*,*B*).

### Deletion of *CCR3* protects against neuronal injury after stroke

**Figure 6.**
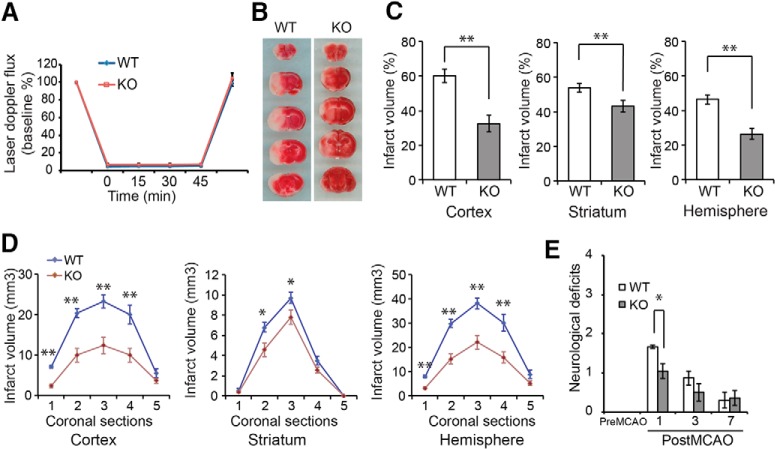
*CCR3* deficiency protects against neuronal injury after stroke. ***A***, Laser-Doppler flux measured over the lateral parietal cortex in the core of the ischemic region in CCR3 KO (n = 13) and WT (n = 9) mice. Values are means ± SEM, expressed as a percentage of pre-ischemic baseline values. ***B***, Representative images of 2,3,5-triphenyltetrazolium chloride (TTC) staining of brain slices from CCR3 KO mice and WT littermate controls subjected to 60 min of MCAO. ***C***, Quantification of infarct volumes in the cortex, hippocampus, and whole hemisphere after 60 min of MCAO in CCR3 KO mice and WT mice. Data are expressed as a percentage of the entire ischemic hemisphere and are the means ± SEM. *p < 0.05 from WT by Student’s *t* test. ***D***, Quantification of infarct volumes among the five coronal levels (level 1 is most anterior) after 60 min of MCAO in CCR3 KO and WT mice. Data represent the mean ± SEM. *p < 0.05 and **p < 0.01 from WT by ANOVA with Tukey–Kramer post hoc test. ***E***, Spontaneous neurobehavioral activity after MCAO was assessed on a scale of 0–4 (0, no neurological deficit; 4, severe neurological deficit) by the criteria described in Methods. Data represent the mean ± SEM, *p < 0.05 from WT by one-way ANOVA with Tukey–Kramer post hoc test.

To extend these studies *in vivo*, WT and CCR3 KO mice were subjected to MCAO. Over the 45-min occlusion, cortical perfusion blood flow monitored by laser-Doppler flowmetry was reduced equivalently in WT (4.88 ± 0.15% of baseline, mean ± SEM) and CCR3 KO (6.74 ± 0.10% of baseline, mean ± SEM) mice. The reduction was stable throughout the occlusion period and recovered to pre-ischemic levels immediately on removal of the filament (Fig. 6*A*). Despite the similar intensity of the insult, infarct volume was reduced in the cortex and striatum and overall in the infarcted hemisphere by 46.12, 19.56, and 43.43% in CCR3 KO mice compared with WT littermates (Fig. 6*B*,*A*). Moreover, the reduction in infarct volume was not skewed to a particular brain region, but was uniformly reduced in CCR3 KO brains (Fig. 6*D*). Neurobehavioral activity was assessed by spontaneous activity in the open-field assay 24 h after MCAO. Similar to the infarct data, CCR3 KO mice have improved neurobehavioral scores compared with WT mice (Fig. 6*E*). These results, taken together, demonstrate that *CCR3* is a mediator for neuronal cell death after ischemic insult.

## Discussion

Neuronal cell death after injury or disease significantly impacts quality of life. Although some neuronal cell death pathways have been uncovered, because of the complexity of the brain it is not inconceivable that there remain additional cell death signaling networks to be discovered. DNA damage is an important contributor to the activation and propagation of neural cell death signaling events. DNA damage is a prominent feature in a number of acute and chronic neurological diseases including stroke, Alzheimer’s disease, Parkinson’s disease, and amyotrophic lateral sclerosis. In the present study, we identified 80 genes that participate in DNA damage–induced cell death by genome-wide screening using an siRNA library. Bioinformatic analysis suggests that these 80 genes are connected to several partially overlapping and interconnected pathways and protein complexes, including negative regulation of catalytic activity, negative regulation of transferase activity, damaged DNA binding, hydrolase activity acting on glycosyl bonds, and negative regulation of MAPK activity. The results provide new insight into neural cell death signaling pathways. Indeed, many of the genes and pathways identified in this screen have not been previously linked to DNA damage–induced cell death, suggesting that the networks that govern neuronal cell death encompass a broad range of cellular functions. The identification of uncharacterized novel genes in this functional screen will provide valuable cues for investigating their individual functions. Six genes encoding protein kinase activity were identified in this screen, consistent with the noted role of protein kinases as fast transducers for triggering the activation of their substrates and initiating cell death pathways (Peng and Chen, 2003; Shiotani and Zou, 2009). In this screen, eight mitochondrial mediators (Ndufb2, Mrpl17, Mosc2, Snap91, Trim39, Nme1, Abce1, and Mtg1) were identified that contribute to cell death, suggesting that both intrinsic and extrinsic mitochondria-mediated cell death signaling pathways play pivotal roles as well. This is consistent with the concept that mitochondria actively participate in neuronal cell death and are important contributors to the pathogenesis of prominent neurodegenerative diseases and psychiatric disorders (Mattson et al., 2008).

CCR3 is a member of the seven transmembrane G protein-coupled receptors that bind C-C type chemokines. CCR3 is activated by a large number of chemokine ligands with varied selectivity and potency, including CCL5, CCL7, CCL11, CCL13, CCL15, CCL24, CCL26, and CCL28 (Daugherty et al., 1996; Struyf et al., 2001; Willems and Ijzerman, 2010; White et al., 2013; Pease and Horuk, 2014). In the periphery, CCR3 plays significant pathogenic roles in the development of inflammation, allergic reactions, and lung fibrosis (Huaux et al., 2005). In the brain, CCR3 has also been identified in microglia, astrocytes, and neurons (van der Meer et al., 2000; Van Der Meer et al., 2001). Expression of CCR3 is elevated in the setting of human immunodeficiency virus infection (van der Meer et al., 2000; Van Der Meer et al., 2001), and elevated expression of CCR3 is observed in reactive microglia and astrocytes around the amyloid deposits in Alzheimer’s disease (Xia et al., 1998). After MCAO in mice, the expression of CCR3 is induced in neurons around the peri-infarct areas (Tokami et al., 2013). In this study, we found that CCR3 plays a crucial role in neuronal injury. CCR3 deletion or inhibition protects neurons from OGD-induced cytotoxicity in primary cortical cultures. How CCR3 is activated in cortical cultures is not known and requires further study. It may promote activation of a cell death cascade through its activation of the MAPK signaling pathway or phospholipase Cγ signaling (White et al., 2013). CCR3 KO mice also displayed significant reduction of infarct volume in the brain after MCAO. These data are consistent with our findings in cortical cultures for a neuronal role of CCR3. However, *in vivo* after MCAO, CCR3 will also be directly activated by elevated chemokines because of the inflammatory response (Perez-Alvarez and Wandosell, 2016). Indeed, the CCR3 ligand, CCL5, is rapidly induced in the brain after MCAO and thus would be able to activate CCR3 (Tokami et al., 2013). Interestingly, knockout of CCL5 has been shown to provide protection against MCAO in mice, although the mechanism under study was the regulation of microvascular dysfunction (Terao et al., 2008). It is possible that local production of CCL5 could also be the mechanism of activation of CCR3 in cortical cultures, which comprise both neurons and glia. Although there is strong evidence for inflammatory mediators expressed and acting within the CNS, in CCR3 KO mice we cannot exclude the possibility that protection is also afforded by inhibition of peripheral inflammatory responses. Taken together, our findings imply a role for CCR3 in neuronal cell death and cell death after ischemic injury in murine models. These initial findings support the need for additional investigations into the actions of CCR3 in neuronal demise to determine whether inhibiting CCR3 could provide a new avenue for the future treatment of ischemia injury or neurodegenerative disease.

In summary, siRNA library screening is a powerful and efficient method to globally identify signaling pathways relevant to neural cell death. Identification of the network of neural cell death mediators will greatly enhance our understanding of the molecular mechanisms of neural cell death and in the future may provide new therapeutic targets for injury to or diseases of the nervous system.

## Synthesis

The decision was a result of the Reviewing Editor Jun Chen and the peer reviewers coming together and discussing their recommendations until a consensus was reached. A fact-based synthesis statement explaining their decision and outlining what is needed to prepare a revision is listed below. The following reviewer(s) agreed to reveal their identity: Ruth Stetler

Both reviewers identified interesting aspects of the current study. In specific, the technique of high content genome-wide siRNA screen provides a useful tool to screen silenced genes under different conditions, with which the authors have identified novel players in cell death downstream of DNA damage in neurons. Based on the gene screening results, the authors attempted to confirm whether one of these critical proteins (CCR3) mediates neuronal ischemic injury, which is demonstrated to involve DNA damage, through use of both in vitro and in vivo models. The finding that knockdown or knockout out of CCR3 led to protection against OGD or transient focal ischemia helps to support the concept that CCR3 contributes to ischemic injury. Despite of the interesting findings, there are several issues about the study that should be addressed by the authors.

Major points:

1. The expression profiles and responses to the injury of neural stem cells should be different from the neurons. The authors used neural stem cells to screen the mediators for DNA damage-induced neuronal cell death in this paper. So were the identified genes specific mediators for neuronal death or just common mediators for cell death of different kinds of cells?

2. The immune cells including neutrophils and macrophages/microglia express CCR3. So CCR3-/- mice showed smaller infarction and less neurological deficits via reducing the neuronal death or regulating the inflammation after stroke? The authors should show some evidences to support their conclusions.

3. In the ischemic models, the authors use either pharmacological pretreatment to inhibit CCR3, or a global CCR3 knockout model followed by ischemic injury. The outcomes are acute cell survival/death (in vitro) or gross histology (TTC, in vivo). No analyses of DNA damage or specific morphology have been performed. This leads to two issues. First, the role of CCR3 in ischemic injury could be completely independent of its initiation downstream of DNA damage, since the paradigm inhibits CCR3 upstream of DNA damage, and thus decouples the MNNG experiments from the ischemia models. Second, a major issue in cell death protection has been repeatedly described in the literature, wherein inhibition of molecular events simply alters morphology or delays cell death. The authors rely on acute timepoints for histology, and do not perform cellular-based histological assays with the in vivo model.

4. The authors claimed that "CCR3 functions as a death receptor to trigger the neuronal cell death pathway during brain ischemia injury". What ligands of CCR3 would initiate this effect, the regular chemokines ligand of CCR3 or other specific ligands? The author should at least discuss the potential mechanisms underlying the roles of CCR3 in cell death.

Minor points:

1. The authors state in the methods the use of siRNA lentiviral transduction, but the results (p. 9) and figure 2A note shRNA. There is a nuance between the two terms - please amend for consistency or accuracy. Secondly, multiple grammatical errors are present throughout the bulk of the paper that should be corrected.

2. In the validation experiments (Fig 2), did lentiviral shRNA transfection per se affect neuronal viability?

3. Fig 1A, what is "s" or "as"?
